# A Case Report of a Syndromic Triad of Persistent Urogenital Sinus, Herlyn–Werner–Wunderlich Syndrome, and Prune Belly Syndrome in a Neonate

**DOI:** 10.1055/a-2806-3084

**Published:** 2026-02-24

**Authors:** Elena Grömping, Johanna Hagens, Hans C. Schmidt, Katharina Wenke, Christian Tomuschat, Konrad Reinshagen

**Affiliations:** 1 Department of Pediatric Surgery, University Medical Center Hamburg-Eppendorf, Hamburg, Germany

**Keywords:** case report, Prune Belly syndrome, persistent urogenital sinus, Herlyn–Werner–Wunderlich syndrome, urinary ascites

## Abstract

**Background:**

We present a case of a premature female neonate with a triad of persistent urogenital sinus with urinary ascites, bilateral hydrocolpos in a duplex uterus, and abdominal wall hypoplasia resembling Prune Belly-like syndrome, combined with severe bilateral cystic dysplastic kidneys and complex urinary obstruction.

**Case Report:**

A female infant was born at 34 ^3/7^ weeks' gestation via cesarean section due to prenatal detection of hydrops fetalis and massive urinary ascites. Prenatal ultrasound had shown oligohydramnios, ascites, megacystis, and hydrocolpos. Postnatally, urinary ascites, a persistent urogenital sinus, severe upper urinary tract dilation, bilateral dysplastic kidneys, and an obstructive vaginal septum were confirmed. Management included staged urinary drainage, vaginal septum incision, intensive respiratory and renal support, and multidisciplinary care.

**Conclusion:**

This unique combination of anomalies presents significant diagnostic and therapeutic challenges. Early recognition and individualized multidisciplinary management are essential to improve postnatal outcomes and guide long-term planning in such cases.

**New Insights and Importance for the Pediatric Surgeon**

To our knowledge, this is the first reported case of a female neonate presenting with the triad of persistent urogenital sinus with urinary ascites, bilateral hydrocolpos in a duplex uterus, and abdominal wall hypoplasia mimicking Prune Belly-like syndrome. The case highlights the importance of early prenatal detection, multidisciplinary management, and tailored surgical approaches to optimize outcomes in such complex congenital anomalies.

## Introduction

Congenital anomalies include a variety of diseases, ranging from more common disabilities like heart defects and anorectal malformations to rarely seen conditions with individual and complex anatomical malformations affecting various organ systems. Treatment of the latter is challenged by limited standardization, and treatment strategies often rely on clinical experience and individual solutions. This report describes the complex case of a premature female infant with simultaneous manifestation of a persistent urogenital sinus with urinary ascites, abdominal aplasia linked with Prune Belly-like syndrome, and urinary tract malformations likely related to a variant of Herlyn–Werner–Wunderlich syndrome. Despite extensive genetic testing, no specific anomalies have been confirmed. Additional conditions, such as bronchopulmonary dysplasia and retinopathy, may result from prematurity. 1 The interrelationship among these anomalies, whether representing a primary syndromic entity or a cluster of independent malformations, remains unclear and warrants further investigation.

With this case report, we would like to contribute to the collective knowledge in the hope that, in the future, we will be able to draw on further published reports to develop initial recommendations for highly complex congenital malformations. Therefore, the objective of this report is to provide a detailed account of the diagnostic, surgical, and multidisciplinary management of this rare triad of congenital anomalies and to highlight lessons for prenatal counseling, perinatal planning, and surgical care.

Ethical approval was not required (Hamburg Medical Association IRB 2025-300599-WF); written parental consent for data and image publication was obtained.

## Case Presentation

### Birth and Initial Care

Prenatal ultrasound at 30 weeks revealed oligohydramnios and progressive abdominal fluid accumulation, suggestive of urinary ascites. Megacystis and bilateral hydrocolpos were also noted. The timing of ascites detection correlated with evidence of fetal urinary tract obstruction. No prenatal intervention (e.g., vesicoamniotic shunting) was performed due to the complex syndromic presentation. The girl, delivered via cesarean section at 34 ^3/7^ weeks of gestational age due to transverse fetal position and prenatal malformations, including hydrops fetalis, weighed 3,860 g (>99th percentile), measured 47 cm (60th percentile), and had a head circumference of 32 cm (48th percentile). She was the mother's first child after three early miscarriages. Her condition was critical at birth, with an APGAR score of 4/7/7 and cord blood pH of 7.14, with signs of respiratory acidosis. Immediate intervention was required for apnea and low oxygen saturation initially, 30%, and a heart rate of 80 bpm. Intubation was done at the fourth minute, stabilizing oxygen saturation to 85% within 30 minutes. An ultrasound revealed a significant left pleural effusion, necessitating chest tube insertion to drain 40 mL of fluid. An umbilical vein catheter was placed for infusion therapy and blood sampling. Blood gas analysis showed worsening respiratory acidosis, leading to initiation of intratracheal surfactant therapy. The infant was transferred to the neonatal intensive care unit for respiratory support and management of prematurity complications.

### Pulmonology and Cardiology

**Fig. 1:**
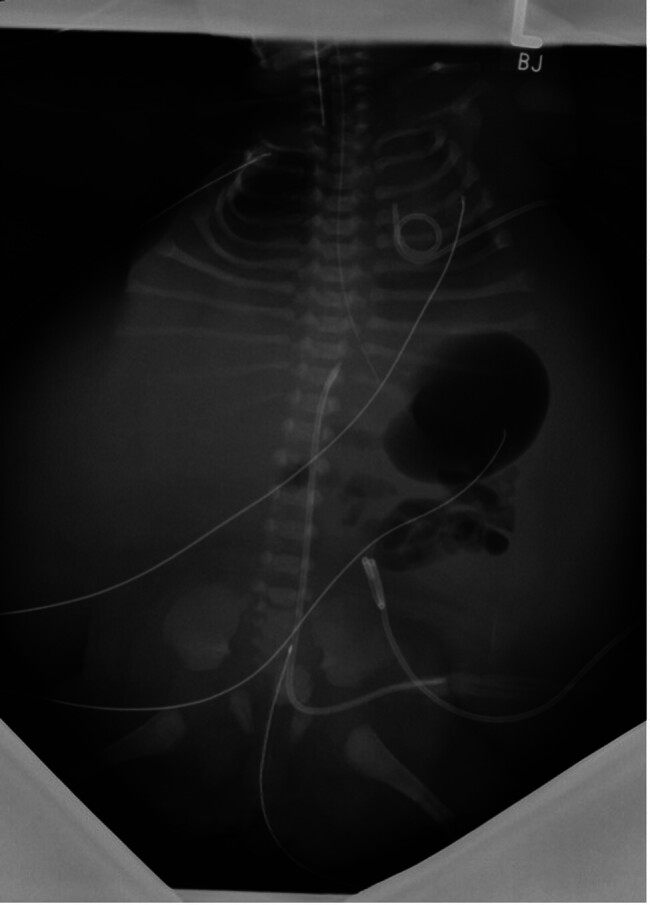
X-ray examination of thorax and abdomen of the infant in anterior–posterior projection on the first day of life. The photograph showed a massive abdominal distension due to urinary ascites, a bell-shaped thorax, an air-filled stomach, as well as proximal intestinal loops.

**Fig. 2:**
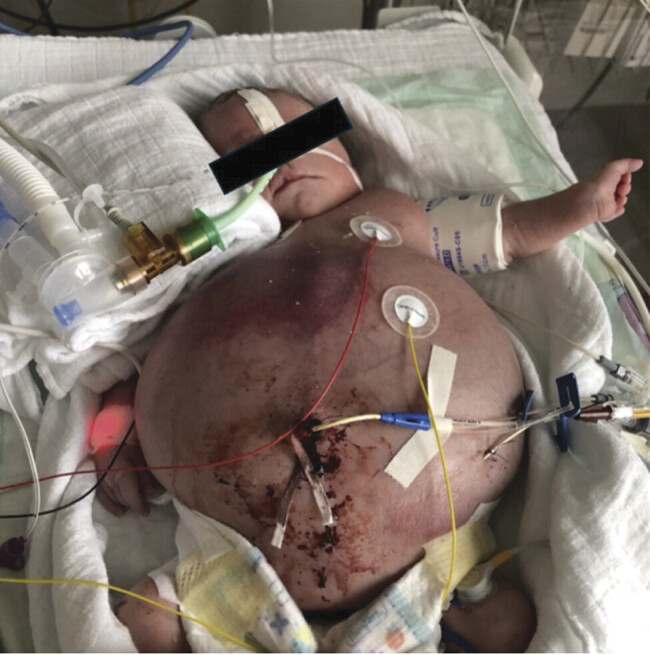
Female premature infant with severely distended abdomen due to urinary ascites. Also visible are the umbilical catheter, the peritoneal drainage, the nasal tube, and ECG electrodes.

Initial chest X-ray ( Fig. 1 ) showed bronchopulmonary dysplasia with ground glass opacities and a bell-shaped thorax. The infant required 21 days of mechanical ventilation, followed by non-invasive support. Respiratory mechanics were impaired by hypotonic muscle tone and urinary ascites ( Fig. 2 ), with mild permissive hypercapnia. Spironolactone was administered to support pulmonary function, along with respiratory syncytial virus prophylaxis. 2 Circulatory instability was managed with catecholamines. An echocardiogram revealed a significant patent ductus arteriosus, successfully closed with paracetamol. Thrombus-like structures along a central venous catheter required heparinization. Sinus tachycardia was treated with propranolol. Multiple hematomas, likely related to intrauterine positioning after anhydramnios, were observed without coagulation abnormalities.

### Urogenital Tract and Renal Function

**Fig. 3:**
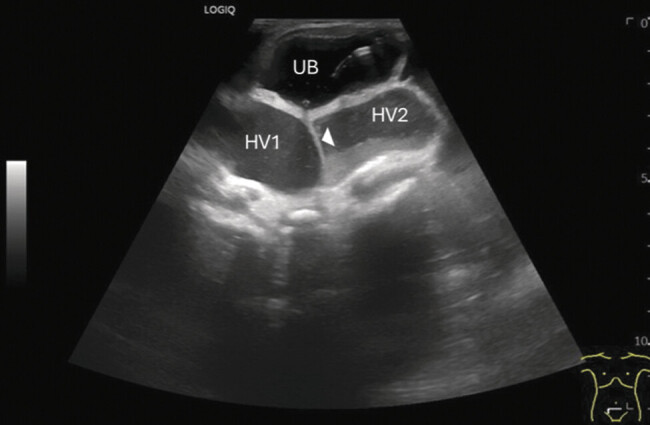
Postnatal ultrasound examination of the urogenital tract showed a distended urinary bladder (UB) with a catheter placed inside and two hemivaginae (HV1, HV2) with hydrocolpos, divided by a septum (◄).

**Fig. 4:**
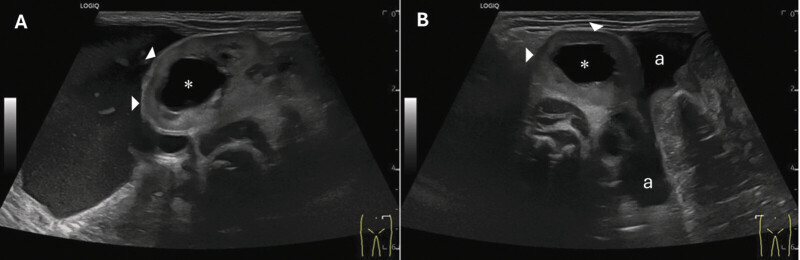
Ultrasound examination of the left ( **A** ) and right kidney ( **B** ). Sonography showed fourth-degree urinary retention (*) with severely displaced renal medulla (▶) and ascites (a) between the intestinal loops in the splenorenal recess (Coller pouch) and hepatorenal recess (Morrison pouch).

Prenatal ultrasound revealed oligohydramnios, compartmentalized ascites, and bilateral urinary retention, indicated by a distended bladder and bilateral hydrocolpos, later confirmed postnatally ( Fig. 3 ). Postnatal examination showed massive abdominal distension and absence of palpable abdominal wall musculature, consistent with Prune Belly-like syndrome. The external genitalia presented with hypertrophic labia and an unlocalizable urethral orifice, while the anus was normally positioned. Sonography suggested a persistent urogenital sinus with cystic structures adjacent to the bladder, consistent with uterus didelphys and hydrocolpos, raising suspicion for Herlyn–Werner–Wunderlich syndrome. Both kidneys exhibited cystic dysplastic changes with severe hydronephrosis and bilateral vesicoureteral reflux, resulting in marked dilation of the ureters and renal pelvis, with hyperechogenic and displaced renal parenchyma ( Fig. 4 ). Bilateral end-diastolic flow disturbances were also observed.

Urinary drainage was initially achieved via suprapubic bladder catheterization and percutaneous drainage of hydrocolpos. Voiding cystourethrography demonstrated bilateral grade IV vesicoureteral reflux with opacification of the hydrocolpos and peritoneal cavity. Serial monitoring of renal function revealed impaired clearance, with an initial cystatin C-based glomerular filtration rate (GFR) of 22 mL/min. Subsequent measurements indicated gradual improvement, with stabilization at 32 mL/min by May 2025. Renal insufficiency led to secondary hyperparathyroidism and anemia, treated with vitamin D, alpha-cholecalciferol, and darbepoetin alpha.

Spontaneous urine output was maintained after decompression. A third-week cystoscopy confirmed the diagnosis of a persistent urogenital sinus. Detection of a short common channel after 1.5 cm of the common channel, the urethra was visualized ventrally. At the level of the bladder neck, both low-lying ureteral orifices were identified, appearing refluxive. Retrograde contrast imaging revealed both dysplastic upper urinary tract systems. The bladder appeared trabeculated but compliant. The vaginal septum was obstructive and required surgical division. At nearly 2 months of age, catheters were placed in each hemivagina, and the vaginal septum was incised to relieve left-sided hydrocolpos. Initial management also involved puncture and drainage of urinary ascites, attributed to obstructive uropathy and reflux of urine and vaginal secretions into the peritoneal cavity, causing chemical peritonitis.

### Gastrointestinal Tract

The infant initially exhibited feeding intolerance and poor weight gain, necessitating parenteral nutrition. A gastric tube was placed to relieve gastrointestinal discomfort and facilitate decompression. At 1 month of age, an intestinal motility disorder was diagnosed and treated with neostigmine. No mechanical intestinal obstruction was detected on contrast-enhanced radiography; motility disturbance was confirmed on functional ultrasound. A Broviac catheter was inserted to enable long-term parenteral nutrition. Persistent hypernatremia was managed by discontinuing additional sodium supplementation. Reduced intestinal motility led to recurrent vomiting and constipation, impeding enteral feeding with breast milk. To optimize bowel function, treatment with Macrogol and Naloxone, combined with rectal irrigations, was initiated. The infant required ongoing parenteral nutrition, though small volumes of oral feeding were gradually introduced. Right lateral positioning improved gastric drainage. By 2 months of age, parenteral nutrition was progressively weaned in favor of physiological infusions. An infusion regimen was initiated at 81 mL per day over 18 hours and subsequently reduced to 12 hours. The infant tolerated approximately 500 mL of the targeted 550 to 650 mL orally, supporting satisfactory weight gain. Minor gastrointestinal findings included echo-rich liver parenchyma, prominent hepatic venous system, possible bile duct dilation, and gallbladder concretions and sludge consistent with non-irritant cholecystolithiasis.

### Infectiology

Infectious complications were managed with staged antimicrobial therapy. Initial treatment with ampicillin and gentamicin was adjusted to cefotaxime due to renal concerns and subsequently escalated to meropenem and teicoplanin in response to rising inflammatory markers. The patient's fluctuating inflammatory profile was attributed to chemical peritonitis, secondary to urinary ascites. No bacterial or viral pathogens were identified in cultures. Prophylactic antibiotics were administered to prevent urinary tract infections associated with indwelling catheters.

### Central Nervous System and Ophthalmology

Cranial ultrasound on the first day of life showed a megacisterna magna, mild ventricular dilation, and a hyperechogenic lesion in the left thalamus suggestive of hemorrhage or ischemia. Subsequent imaging identified a small plexus cyst without hydrocephalus. Neurological examination initially revealed generalized hypotonia and weak reflexes. Ophthalmological evaluation diagnosed bilateral cataracts, iris colobomas, and vitreous anomalies consistent with persistent fetal vasculature; both eyes were treated with lens aspiration and vitrectomy, followed by referral for visual rehabilitation.

### Genetic Assessment

Given the complex constellation of congenital anomalies, an underlying genetic etiology was suspected. 3 The patient, first child of unrelated parents after three early miscarriages, underwent trio exome sequencing. 4 No pathogenic variants were identified, including for known syndromes such as Noonan and Prune Belly-like syndrome. 5 Dysmorphic features and the combination of urogenital anomalies suggested a possible variant of Herlyn–Werner–Wunderlich syndrome. 6 Further postnatal trio exome analysis is ongoing.

## Discussion

The following discussion explores the embryological and clinical context of the patient's anomalies and addresses key considerations for diagnosis, management, and prognosis.

### Urogenital and Renal Anomalies: Surgical Considerations

This case illustrates the diagnostic and surgical complexity of managing a rare constellation of persistent urogenital sinus, bilateral hydrocolpos in a duplex uterus, bilateral cystic dysplastic kidneys, severe vesicoureteral reflux, urinary ascites, and abdominal wall hypoplasia (Prune Belly-like syndrome).

Urinary outflow obstruction through the persistent urogenital sinus led to severe bilateral hydronephrosis, hydrocolpos, and progressive urinary ascites. These anomalies were identified prenatally on ultrasound and confirmed postnatally. The presence of urinary ascites was an important finding, as prenatal urinary leakage into the peritoneum may help preserve renal function in obstructive uropathy by decompressing the urinary tract. 7 In this case, however, urinary ascites contributed significantly to respiratory compromise and abdominal distension at birth. 8 9

Initial management focused on rapid decompression of the obstructed urinary tract and hydrocolpos. A suprapubic bladder catheter and bilateral transvaginal catheters were placed, resulting in improved respiratory mechanics and reduction of abdominal girth. Ascites was drained percutaneously and was found to consist of urine and vaginal secretions, consistent with chemical peritonitis. 10 VCUG demonstrated bilateral grade IV vesicoureteral reflux with opacification of the hydrocolpos and leakage into the peritoneal cavity. 9

Cystoscopy revealed a short common urogenital channel (approximately 15 mm), with a high confluence of urethra and vagina and ectopic ureteral orifices entering the common channel. 10 A thick, obstructive vaginal septum was identified and surgically divided at nearly 2 months of age to achieve adequate bilateral drainage. Persistent spontaneous urine output was observed following decompression, and serial monitoring of renal function showed gradual improvement (initial cystatin C clearance 22 mL/min; latest 32 mL/min; serum creatinine 0.45 mg/dL).

The presence of uterus didelphys with bilateral hydrocolpos and ipsilateral and contralateral renal dysplasia suggested a variant of Herlyn–Werner–Wunderlich syndrome. 9 However, given the absence of laparoscopic confirmation and the complex overlapping anomalies, this diagnosis remains presumptive.

Abdominal wall hypoplasia consistent with Prune Belly-like syndrome was also present and contributed to impaired respiratory and gastrointestinal function. In this female patient, classical Prune Belly syndrome (which, by definition, includes cryptorchidism and is almost exclusively male) cannot be diagnosed. 11 12 We therefore refer to this presentation as “Prune Belly-like syndrome” or abdominal wall hypoplasia associated with urinary obstruction. 13

Comparable cases in the literature underscore the importance of early multidisciplinary intervention. Wolf et al 10 and Nigam et al 14 reported similar cases of urogenital sinus with fetal urinary ascites, successfully managed by staged surgical repair. Yamamichi et al 8 emphasized the need for individualized perinatal planning in cases of persistent cloaca and urinary ascites. Our experience aligns with these observations and highlights the critical role of early urinary drainage in optimizing outcomes.

Given the high risk of progressive renal impairment and potential future surgical needs, close multidisciplinary follow-up is essential for this patient. 15

### Outlook

Assessing the prognosis for this patient remains challenging due to the complexity of her anomalies. Each condition carries a high risk of morbidity, and their combined presentation requires ongoing intensive management. 9 16 While early surgical decompression and multidisciplinary care have stabilized her clinical course, long-term risks include progressive renal impairment, potential continence and reproductive challenges, and neurodevelopmental impact. 17 Given the limited follow-up to date, statements regarding future menstruation, fertility potential, or adult quality of life must remain speculative. Close multidisciplinary follow-up will be essential to monitor renal function, urological outcomes, and neurodevelopment, and to guide future surgical planning and transitional care. 18

## Conclusion

This case illustrates the diagnostic and therapeutic complexity of managing a rare combination of persistent urogenital sinus, bilateral hydrocolpos, abdominal wall hypoplasia, and severe urological anomalies. Early prenatal detection, prompt decompression of obstructive lesions, and individualized multidisciplinary management were essential to stabilizing the patient. This experience underscores the need for collaborative surgical planning and long-term follow-up in managing such rare congenital anomalies.
